# A 1D Model for Predicting Heat and Moisture Transfer through a Hemp-Concrete Wall Using the Finite-Element Method

**DOI:** 10.3390/ma14226903

**Published:** 2021-11-16

**Authors:** Maroua Benkhaled, Salah-Eddine Ouldboukhitine, Amer Bakkour, Sofiane Amziane

**Affiliations:** 1 Institut Pascal, Université Clermont Auvergne, CNRS, SIGMA Clermont, F-63000 Clermont-Ferrand, France; benkhaledmarwa.147@gmail.com (M.B.); salah_eddine.ouldboukhitine@uca.fr (S.-E.O.); 2 Department of Physics, Faculty of Sciences, Lebanese University, Hadath, Beirut, Lebanon; amer.bakkour.95@gmail.com

**Keywords:** hemp concrete, heat, air and mass transfer, numerical implementation, finite-element method

## Abstract

Plant-based concrete is a construction material which, in addition to having a very low environmental impact, exhibits excellent hygrothermal comfort properties. It is a material which is, as yet, relatively unknown to engineers in the field. Therefore, an important step is to implement reliable mass-transfer simulation methods. This will make the material easy to model, and facilitate project design to deliver suitable climatic conditions. In recent decades, numerous studies have been carried out to develop models of the coupled transfers of heat, air and moisture in porous building envelopes. Most previous models are based on Luikov’s theory, considering mass accumulation, air and total pressure gradient. This theory considers the porous medium to be homogeneous, and therefore allows for hygrothermal transfer equations on the basis of the fundamental principles of thermodynamics. This study presents a methodology for solving the classical 1D (one-dimensional) HAM (heat, air, and moisture) hygrothermal transfer model with an implementation in MATLAB. The resolution uses a discretization of the problem according to the finite-element method. The detailed solution has been tested on a plant-based concrete. The energy and mass balances are expressed using measurable transfer quantities (temperature, water content, vapor pressure, etc.) and coefficients expressly related to the macroscopic properties of the plant-based concrete (thermal conductivity, specific heat, water vapor permeability, etc.), determined experimentally. To ensure this approach is effective, the methodology is validated on a test case. The results show that the methodology is robust in handling a rationalization of the model whose parameters are not ranked and not studied by their degree of importance.

## 1. Introduction

Today, there is growing interest in the problem of coupled transfer of heat, air, and moisture (HAM) in porous building materials. The coupled heat and mass transfer processes have a significant impact on building performance in terms of indoor comfort, energy use to heat and cool the space, and envelope durability. Where the building envelopes contain air-filled cavities, it becomes more complex to describe the heat and mass transfer phenomena, which depend on the thermal convective movements of air. Therefore, in construction, in order to better predict the temperature and hydric behavior of porous building walls, an accurate assessment of the coupled HAM transfer through the envelope is required. So too is an assessment of the dynamic hygrothermal interaction between the external environment and the building’s indoor air. To achieve optimal performance, numerical models have been developed to simulate the hygrothermal response of building envelopes. The analysis of the energy performance of building materials requires a simple numerical tool to predict their hygrothermal behavior.

In order to predict the hygrothermal behavior of building materials, many phenomenological models of transfers have been developed, and many numerical tools released. Concerning the transport of moisture, but making no reference to that of air, Luikov (1964) [1] developed phenomenological models to describe transport in unsaturated porous media, including the effects of temperature. The theory of Luikov considers the porous medium to be homogeneous, and therefore allows for hygrothermal transfer equations on the basis of the fundamental principles of thermodynamics [2].At the scale of a real-life building, Glaser’s method [3] was the first procedure for evaluating vapor diffusion transport in a one-dimensional steady state, without considering moisture and heat accumulation in the materials.

Considering liquid flow, Kunzel and Kiessl (1997) [4] developed more complete models taking the liquid and vapor diffusive transport into account. They used WUFI software to solve the non-linear transport equations. Over the past twenty years, many mathematical models and numerical methods applying to different fields have been described [5,6,7,8,9,10,11].

Considering heat transport, many studies have been conducted to understand the thermal properties of porous materials. However, Al-Hazmy (2006) [12] studied the coupled convective–conduction effects on the mode of heat transport in a hollow building brick. The study showed that the effect of natural intercellular convection on the thermal resistance of hollow structures cannot be ignored. The FLUENT CFD software was used to solve the coupled equations.

Various approaches in the literature address coupled HAM transport. Dos Santos and Mendes (2008) [13] proposed a mathematical model considering the combined two-dimensional transport of heat, air, and moisture through unsaturated hollow building bricks. The differential equations are based on the driving potentials of temperature, moist air pressure and water vapor pressure gradients. The discretized equations are solved using the MTDMA (multitridiagonal-matrix algorithm). Li et al. (2009) [14] developed a combined model of heat, air, and moisture in building envelopes (HAM-BE) by using COMSOL software to solve the governing partial differential equations. Belleudy et al. (2016) [15] developed a numerical 2D model, HAM-Leakage, to simulate the coupled heat, air, and moisture transfers through combined porous media and air channels. The developed model is implemented in COMSOL Multiphysics. Bennai et al. (2016) [16] developed a macroscopic model of hygrothermal transfers in porous building materials using multi-scale periodic homogenization. The problem is solved by the finite-element method, using COMSOL Multiphysics. Škerget et al. (2017) [17] formulated the boundary element numerical model (BEM) to solve the two-dimensional time-dependent coupled non-linear HAM flow through a porous solid. Ayres de Mello et al. (2019) [18] proposed a new mathematical model, called CAR-HAM (conductive, advective, and radiative heat, air and moisture), including the radiative transfer equation to calculate the thermal radiation effects within porous building materials. The equations are simultaneously solved using the MTDMA.

Considering coupled HAM transfer problems, the biggest difference between the simulated models is the choice of solving methodology for the HAM model. In the previous literature, there has been no fully virtual evaluation of the hygrothermal behavior of porous building material. Most simulation tools act as black boxes, including a library of pre-defined blocks to be used to construct and simulate the models. The disadvantage of these methods is that, often, they are quite inflexible in terms of changing or combining models. For this reason, in order to improve the simulation performance, this article presents the finite element method implemented on MATLAB software to solve the HAM hygrothermal transfer model. This software provides high-level visualization of parameters, good algorithm development and a wide range of programming freedom.

The objective of this article is to study and analyze the hygrothermal behavior of hemp concrete. In order to do this, it is necessary to consider coupled heat, air, and mass transfers throughout all components of the envelope. The model considers the temperature, the total pressure, and the water vapor pressure gradients as driving potentials. Based on this model, a one-dimensional numerical simulation is carried out. The results have been simultaneously obtained using MATLAB. Numerical validation of this method is presented, based on experimental results and published numerical benchmarks. The MATLAB model offers more programming freedom and is thus more closely suited to bio-based material studies. In fact, the hygrothermal properties of hemp concrete are not constant; they vary depending on several factors such as the evolution of water content within the wall. Therefore, MATLAB would be more efficient to code these properties. The model proposed in the present paper gives the freedom to vary these parameters with ease. It was also developed in such a way as to make the future work (sensitivity analysis of parameters) accessible and reliable.

## 2. Developed Model

We have compiled an overview of the phenomenological models developed by numerous researchers. These models are based on the establishment of equations reflecting coupled HAM transfer through porous walls. Most transfer models available in the literature consider the porous material as a black box. The difference between these models lies in the choice of driving potentials. For energy transfer, temperature is an often-used transfer potential. On the other hand, concerning mass transport, there are several hydric driving potentials. According to Funk and Ghazi [19], these models would produce similar results if specific assumptions such as local thermodynamic equilibrium are taken into account. In short, the appropriate choice of driving potentials is not always evident. Water content is not a state variable in the thermodynamic sense. Using vapor pressure avoids discontinuity at the interfaces between layers of the wall.

### 2.1. Hypotheses

In order to simplify the modeling of coupled HAM transfer, the following hypotheses have been adopted:

- At any point of the unsaturated porous-continuum, the different phases (solid, liquid and gas) are in local thermodynamic equilibrium;
- The solid medium is non-deformable and homogeneous;
- The gas phase obeys the ideal gas law;
- Heat transfer by radiation is negligible;
- Moisture transfer due to gravity is negligible.

### 2.2. Coupled Transfer Governing Equations

Firstly, the mass and energy conservation equations are described for all phases. Then they are defined as a function of the transfer driving potentials given by temperature, vapor pressure and air pressure gradients. The energy and mass balance equations are defined for each phase present. The local balance equations of the liquid phase (Equation (1)), of the vapor phase (Equation (2)), the dry air balance (Equation (3)) and the energy balance (Equation (4)) are as follows:

(1) $$\frac{\partial u_{l}}{\partial t}=-div(j_{l})$$

(2) $$\frac{\partial u_{v}}{\partial t}=-div(j_{v})$$

(3) $$\frac{\partial u_{a}}{\partial t}=-div(j_{a})$$

(4) $$c\rho_{s}\frac{\partial T}{\partial t}=-div(j_{q})$$

where $u_{l}$, $u_{v}$, and $u_{a}$ [kg/m^3^] are the contents of liquid water, vapor and dry air, respectively. They are also given by the relation $u=\omega \cdot \rho_{s}\cdot j_{i}$ [kg/(m^2^.s)] is the mass flow density of phase *i* (*i* = *v* for the water vapor phase, *i* = l for the liquid phase and *i* = a for the dry air phase), $\rho_{s}$ [kg/m^3^] is the dry density of the sample, $c_{p}$ [J/(kg.K)] is the heat capacity of the material, $j_{q}$ [J/(m^2^.s)] is the heat flux density, *h_lv_* [J/kg] is the enthalpy, ω [kg/kg] is the water content, and T [K] is the temperature.

According to Crausse, Laurent and Perrin [20], heat transfer is attributable to three factors: the pure conduction heat transfer given by Fourier’s law, the convective transfer of tangible heat by liquid and vapor flows, and the transfer of latent heat by vapor. Therefore, the heat flux density is expressed by:

(5) $$j_{q}=-\lambda \nabla T+h_{l}j_{l}+h_{v}j_{v}$$

where $h_{l}$ [J/kg] is the enthalpy of liquid water, $h_{v}$ [J/kg] is the enthalpy of water vapor, and λ [w/(m.K)] is the thermal conductivity.

#### 2.2.1. Vapor Phase Transfer

Macroscopic moisture transfer in the vapor phase is described by Luikov [1], as a diffusion expression suited to Fick’s law, expressed by the first term in Equation (6). If, during the vapor transfer, there is a total pressure gradient, it creates an additional molar transfer described by Darcy’s law. It is represented by the second term in the vapor flux density expression in Equation (6). In this kind of transfer, the expansion of the air bubbles forces the vapor in the direction of heat flow under the pressure gradient. Thus, the air pressure in the small pores increases with increasing heat, creating “infiltration” movements. In this case, the form of the macroscopic diffusion law is identical to the microscopic law.

(6) $$j_{v}=-k_{v}\nabla P_{v}-k_{fv}\nabla P$$

where $j_{v}$ [kg/(m^2^.s)] is the mass flow density of the vapor, and *k_fv_* [kg/(m.s.Pa)] is the molar filtration coefficient of the vapor. This parameter is the result of two components: the capillary surface and the hydraulic capillary conductance. $P_{v}$ [Pa] is the vapor pressure, $k_{v}$ [kg/(m.s.Pa)] is the water vapor permeability in air multiplied by a factor representing the permeability resistance. *P* [Pa] is the total pressure (vapor pressure + dry air pressure).

#### 2.2.2. Liquid Phase Transfer

Conventionally, liquid transport is described in macroscopic terms by Darcy’s law. It occurs by capillary absorption under the influence of capillary potentials (due to curved menisci of the gas–liquid interface) and adsorption potential (due to adhesive forces) which are summarized by a capillary suction gradient:

(7) $$j_{l}=-\rho_{l}k\cdot \nabla \psi$$

where $j_{l}$ [kg/(m^2^.s)] is the mass flow density of the liquid phase, *Ψ* [m] is the capillary suction, *k* [m/s] is the hydraulic conductivity, and $\rho_{l}$ [kg/m^3^] is the density of the liquid phase.

In the same way as the vapor phase, a total pressure gradient also causes filtration transfer of the liquid. Equation (7) then becomes:

(8) $$j_{l}=-k_{l}\cdot \nabla P_{c}-k_{fl}\nabla P_{}$$

where $j_{l}$ [kg/(m^2^.s)] is the mass flow density of the liquid phase, *P_c_* [Pa] is the capillary suction, $k_{fl}$ [kg/(m.s.Pa)] is the liquid filtration coefficient, and $k_{l}$ [kg/(m.s.Pa)] is the hydraulic conductivity.

Based on the hypothesis of local equilibrium between the liquid and gas phases in a pore, the capillary pressure takes the following form according to Kelvin’s law [21]:

(9) $$P_{c}=\frac{RT\rho_{l}}{M}\ln(\frac{P_{v}}{P_{v_{sat}}})$$

where $P_{v_{sat}}$ [m^2^/s] is the saturation vapor pressure, *R* [J/(mol.K)] is the perfect gas constant, *M* [kg/mol] represents the molar mass of water, and *T* [K] is the temperature.

Still based on Kelvin’s law, the capillary pressure gradient is written as two combinations of vapor pressure gradient and a temperature gradient, with the introduction of a coefficient representing the contribution of a temperature difference to the transfer of liquid water [22]:

(10) $$\nabla P_{c}=\left[\frac{RT\rho_{l}}{M}\frac{1}{P_{v_{}}}\right]\nabla P_{v_{}}-\left[\frac{RT\rho_{l}}{M}\left(\frac{\partial \ln(\frac{P_{v}}{P_{v_{sat}}})}{\partial T}\right)+\frac{R\rho_{l}}{M}\ln(\frac{P_{v}}{P_{v_{sat}}})\right]\nabla T$$

By substituting Equation (10) back into Equation (8), we will obtain:

(11) $$j_{l}=-k_{l}\cdot \left[\left[\frac{RT\rho_{l}}{M}\frac{1}{P_{v_{}}}\right]\nabla P_{v_{}}-\left[\frac{RT\rho_{l}}{M}\left(\frac{\partial \ln(\frac{P_{v}}{P_{v_{sat}}})}{\partial T}\right)+\frac{R\rho_{l}}{M}\ln(\frac{P_{v}}{P_{v_{sat}}})\right]\nabla T\right]-k_{fl}\nabla P_{}$$

The mass flow density of the liquid phase then becomes:

(12) $$j_{l}=-k_{lv}\nabla P_{v}-k_{T}\nabla T-k_{fl}\nabla P$$

where $k_{lv}=\left[\frac{RT\rho_{l}}{M}\frac{1}{P_{v}}\right]k_{l}$ [kg/(m.s.Pa)] is the liquid water conductivity due to water vapor pressure gradient and $k_{T}=k_{l}\left[\frac{RT\rho_{l}}{M}\left(\frac{\partial ln\left(\frac{P_{v}}{P_{v_{sat}}}\right)}{\partial T}\right)+\frac{R\rho_{l}}{M}ln\left(\frac{P_{v}}{P_{v_{sat}}}\right)\right]$ [kg/(m.s.K)] is the liquid water conductivity due to a thermal gradient.

The liquid and vapor transfers in the hygroscopic region are described by the same law. They are influenced by the vapor pressure gradients $\nabla P_{v}$, temperature gradients ∇T and the total pressure ∇P. Here, the hypothesis of local equilibrium comes into play, meaning we can express the flows $j_{l}$ and $j_{v}$ by means of the gradient of the same intensive quantity *P_v_*, *T* or *P*. Therefore, the liquid and vapor phase transfers can be combined by adding together Equations (6) and (8).

Also, if we accept the assumption that the vapor phase and liquid phase flows are additive, the global moisture flow is written: $j_{m}=j_{l}+j_{v}$

(13) $$j_{m}=-k_{m}\cdot \nabla P_{v}-k_{T}\nabla T_{}-k_{f}\nabla P_{}$$

where *k_m_* = *k_v_* + *k_lv_* and *k_f_* = *k_fl_* + *k_fv_*

The law of mass transfer becomes:

(14) $$C_{m}\rho_{s}\frac{\partial P_{v}}{\partial t}=-div(-k_{m}\cdot \nabla P_{v}-k_{T}\nabla T_{}-k_{f}\nabla P_{})$$

where Cm=(1Pvsat)·(∂ω∂φ) [kg/kg.Pa] is the moisture storage capacity of the material and φ [%] represents the relative humidity (RH), which can also be determined by calculating the slope of the sorption isotherm.

#### 2.2.3. Gas Phase Transfer (Dry Air + Water Vapor)

A total pressure gradient in the hygroscopic material causes a molar transfer of vapor and dry air, depending on the type of filtration. This molar transfer of water vapor is described by the following formula by [1]:

(15) $$j_{a}+j_{v}=-k_{f}\nabla p$$

Equation (12) is fed into the pressure equilibrium equation, which can be simplified by addition of the differential Equations (2) and (3):

(16) $$\frac{\partial \left(u_{a}+u_{v}\right)}{\partial t}=\frac{\partial \left(\rho_{s}\omega_{g}\right)}{\partial t}=-div\left(j_{a}+j_{v}\right)$$

where $\omega_{g}=\omega_{a}+\omega_{v}=\varepsilon \frac{\rho_{g}}{\rho_{s}}\left(1-S_{l}\right),\rho_{g}=\frac{PM}{RT}\;\mathrm{and}\;\varepsilon \cdot S_{l}=\frac{\rho_{s}}{\rho_{l}}\omega_{l}$.

Therefore, the partial derivation of Equation (13) will take the form:

(17) $$\frac{\partial (\varepsilon \rho_{g}\left(1-S_{l}\right)}{\partial t}=\frac{\partial \left(\frac{PM}{RT}\varepsilon \left(1-S_{l}\right)\right)}{\partial t}=div\left(k_{f}\nabla P\right)$$

Thus, dry air transport is described as a function of pressure, temperature and liquid water saturation:

(18) $$\varepsilon \rho_{g}(1-S_{l})=\frac{PM}{RT}\varepsilon \left(1-S_{l}\right)$$

where ε is the porosity of the material, $S_{l}$ is the degree of water saturation, and M [kg/mol] is the molar mass of water.

For a closed system, differentiating Equation (18):

(19) $$d\left(\frac{PM}{RT}\varepsilon \left(1-S_{l}\right)\right)=\frac{\left(1-S_{l}\right)M\varepsilon }{RT}dP-\frac{MP\varepsilon \left(1-S_{l}\right)}{RT^{2}}dT-\frac{PM\varepsilon }{RT}dS_{l}$$

The contributions of the factors dT and $dS_{l}$ are neglected (supposing that the contribution $T^{2}\ll \frac{\left(1-S_{l}\right)M\varepsilon }{RT}$ and $T>S_{l}$). Denoting the ratio $\frac{\left(1-S_{l}\right)M\varepsilon }{RT}$ by $C_{a}$ [s^2^/m^2^] which represents the humid air capacity, the gas phase equation (Equation (17)) then becomes:

(20) $$C_{a}\frac{\partial P}{\partial t}=div\left(K_{f}\nabla p\right)$$

#### 2.2.4. Heat Transfer

Based on the above descriptions and replacing the formulation of heat flow, liquid and vapor flows in the balance equations, the energy equation becomes:

(21) $$c_{p}\rho_{s}\frac{\partial T}{\partial t}=div(\lambda \nabla T+h_{l}j_{m}+h_{v}j_{v})$$

where $h_{l}$ = $C_{l}$(T-T_ref_) and *h_v_* = $C_{l}$(T-T_ref_) + *L_v_*, where *L_v_* [J/kg] is the latent heat of vaporization, $C_{l}$ [J/kg.K] is the mass heat capacity, and T_ref_ [K] is the reference temperature.

Substituting the total mass flow and water vapor densities (Equation (13) and Equation (6) respectively) back into the energy balance equation, the equation becomes:

(22) $$c_{p}\rho_{s}\frac{\partial T}{\partial t}=div(\lambda \nabla T+\alpha \nabla P_{v}+\gamma \nabla P)+L_{v}C_{m}\sigma \frac{\partial P_{v}}{\partial t}$$

where $\alpha =h_{l}k_{m}$ [m^2^/s], which represents the heat transfer coefficient by advection due to the water vapor pressure gradient. The ratio representing the mass exchange of water vapor and the total mass exchange is given by σ=div(jv)div(jm) [–].

Based on the above assumptions, the following mathematical Equations (14), (20) and (22) can be considered as equations for coupled HAM transfers in porous building materials:

$$C_{m}\rho_{s}\frac{\partial P_{v}}{\partial t}=div\left[K_{m}\nabla P_{v}+K_{T}\nabla T+K_{f}\nabla P\right]$$

$$C_{a}\frac{\partial P}{\partial t}=div\left(K_{f}\nabla P\right)$$

$$C_{p}\rho_{s}\frac{\partial T}{\partial t}=div\left(\lambda \nabla T+\alpha \nabla P_{v}+\gamma \nabla P\right)+L_{v}\rho_{s}\sigma C_{m}\frac{\partial P_{v}}{\partial t}$$

It is a system of three strongly coupled partial-differential equations. The transfer driving potentials (*T*, *P_v_*, *P*) used in this HAM formulation are state variables that do not depend on the microstructure of the materials. This justifies the hypothesis of continuity of these values at the interfaces between the layers (such as in multilayered walls). Moreover, the main physical parameters ($\lambda ,\;C_{m},\;k_{m},$ etc.) are non-linear and depend on the water content and the temperature.

In conclusion, this phenomenological study [22] gives a full account of the various phenomena involved in the coupled transfers of heat, air, and mass.

## 3. Numerical Simulation

In the previous section, we presented the numerical model of hygrothermal transfers in a simple wall, which will be adopted later in our study. In this section, the simulation tool and the resolution method of the HAM model presented above will be discussed.

### 3.1. MATLAB Numerical Simulation Tool

The complexity of the coupled thermo-aeraulic transfer equation system makes it impossible to solve analytically. Therefore, numerical resolution tools must be used. Simulation tools differ depending on the resolution method and the final objective. However, the HAM model was first implemented on COMSOL; since the form of the equations and the algorithm of resolution are predefined, we merely need to input the coefficients of the model. However, because COMSOL presents it as a black box, it is far from easy to intervene in the resolution process. On the other hand, MATLAB allows better visualization of the parameters and offers greater programming freedom for algorithm development.

### 3.2. Finite-Element Discretization

**Figure 1:**
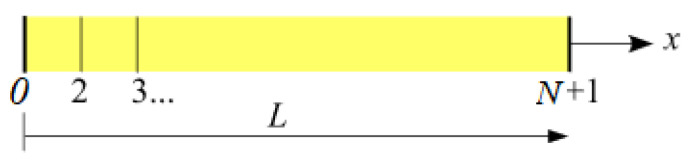
Discretization of a wall.

The system of three equations previously established is discretized by the finite-element method, which uses the θ-scheme. This method can be used to solve non-linear differential equation systems. For this purpose, we use spatial and temporal discretization. The wall is discretized into N elements (Figure 1). Thus, ∆x and ∆t respectively represent the discretization steps of space and time. Note that i denotes the number of nodes and t the instant of calculation.

Within the framework of this treatment, an approximate solution of the problem, using the weighted residual method in Galerkin’s formulation [23], has been adopted. Firstly, we focus on the heat equation. By spatially approximating the solution field of temperatures *T*(*x*,*t*) and on the basis (interpolation) of functions *N*(*x*), the temperature field can be written in the following form:

(23) $$T\left(x,t\right)=\overset{n}{\underset{i=0}{\sum }}\;N\left(xi\right)Ti\left(t\right)$$

The system is solved matricially, so we can obtain the matrix form of the spatially approximated problem:

(24) [C]{T}+[K]{T}={F}

[C] is the matrix of thermal capacity and [K] is the matrix of conductivity. These two matrices are obtained from a concatenation of the elementary matrices defined in each discretization element [Ce] and [Ke], respectively.

**Figure 2:**
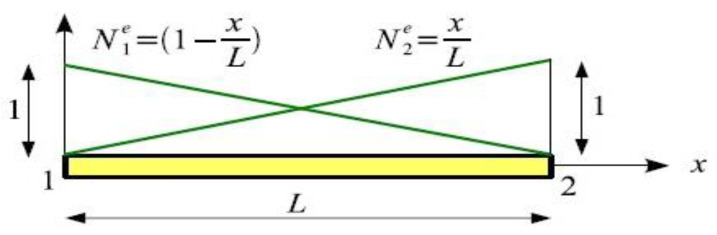
Interpolation functions on a 2-node bar element.

For interpolation functions (Figure 2): $N_{1}=1-\frac{x}{L}$ and $N_{2}=\frac{x}{L}$.

The elementary matrices are calculated as follows:

(25) $$Ce_{ij}=\int_{l}\rho C_{p}N_{i}\left(x\right)N_{j}\left(x\right)dx=\rho C_{p}\int_{l}(1-\frac{x}{L})\left(\frac{x}{L}\right)dx=\rho C_{p}l\left[\begin{array}{cc} \frac{1}{3} & \frac{1}{6}\\ \frac{1}{6} & \frac{1}{3} \end{array}\right]$$

(26) $$Ke_{ij}=\int_{l}\lambda \frac{dN_{i}\left(x\right)}{dx}\frac{dN_{j}\left(x\right)}{dx}dx=\rho C_{p}\int_{l}(-\frac{1}{l})\left(\frac{1}{l}\right)dx=\frac{\lambda }{l}\left[\begin{array}{cc} 1 & -1\\ -1 & 1 \end{array}\right]$$

With respect to the boundary conditions, we apply the Dirichlet conditions by applying temperature differentials at the wall sides. The inside and outside of the building walls represent indoor air environments and outdoor atmospheric environments, respectively. The temperature and humidity fields of a wall are greatly affected by its internal and external environments. In our case, coupled heat and mass equations were solved for boundary conditions corresponding exactly to those of the experimental setup.

The Dirichlet conditions are used to write the equation between the ambient values of temperature, humidity, and pressure, whereas the Neumann conditions take surface exchanges into account. The Neumann conditions may be more realistic for the case of HAM transfers. Based on Oumeziane [24], the simulated distributions of relative humidity and temperature were compared to the experimental distributions within hemp concrete wall, under two sets of conditions: Dirichlet and Neumann conditions. The numerical results obtained under Neumann conditions are very similar to those obtained under Dirichlet conditions as regards relative humidity distributions. Therefore, the choice to work with Dirichlet conditions is preferable since they are more practical to program compared to Neumann conditions, which require special treatment for the finite-element discretization.

This induces a partition of the matrices [C] [K] and vectors {T} ({F} is 0 because there are no flux-type solicitations according to the degree of freedom of the nodes in unknown *T_L_* and known *T_P_* temperatures. Therefore, the heat equation is rewritten in the form (Equation (27)):

(27) $$\left[\begin{array}{cc} C_{LL} & C_{LP}\\ C_{PL} & C_{PP} \end{array}\right]\left\{\begin{array}{c} \dot{T_{L}}\\ \dot{T_{N}} \end{array}\right\}+\left[\begin{array}{cc} K_{LL} & K_{LP}\\ K_{PL} & K_{KP} \end{array}\right]+\left\{\begin{array}{c} \dot{T_{L}}\\ \dot{T_{N}} \end{array}\right\}=\left\{\begin{array}{c} F_{L}\\ F_{N} \end{array}\right\}$$

Once the development is done, Equation (27) can be written as:

(28) $$\left[C_{LL}\right]\{{\dot{T}}_{L}\}+[K_{LL}]\{T_{L}\}=-[C_{LP}]\{{\dot{T}}_{P}\}-[K_{LP}]\{T_{P}\}$$

Integrating a temporal integration ϴ-scheme, to decompose the temperature as follows: {*T_L_*(*t*)} = (1 − ϴ) {*T_L_*}*_n_* + ϴ{*T_L_*}*_n_*_+1_. Let ∆*T_L_* = {*T_L_*}*_n_*_+1_-{*T_L_*}*_n_*, then we have {*T_L_* (*t*)} = {*T_L_*}*_n_* + ϴ {∆*T_L_*}.

Otherwise, {${\dot{T}}_{L}$} = $\frac{\{\mathrm{TL}\}n+1-\{\mathrm{TL}\}n}{∆t}$ = $\frac{\{∆\mathrm{TL}\}}{∆t}$. Replacing {*T_L_*(*t*)} and {${\dot{T}}_{L}$} in the equation, we obtain:

(29) $$\{∆T_{L}\}[C_{LL}]+∆t\theta [K_{LL}]=∆t[-[C_{LP}]\{{\dot{T}}_{P}\}-[K_{LP}]\{T_{P}\left(t\right)\}-[K_{LL}]\{T_{L}\left(t\right)\}]$$

If the boundary conditions are constant, {${\dot{T}}_{P}$} = 0.

After imposing an initial condition in temperature type {*T_L_* (*t* = 0)}, we can then calculate the {∆*T_L_*} for each time step and then work back to the value of {*T_L_*}_n+1_. Through this approach, we rewrite the HAM equations in MATLAB as follows:

(30) $$\{∆T\}[[C_{LL1}]+∆t\theta [K_{LL1}]]=∆t[-[K_{LP1}]\{T_{P}\left(t\right)\}-[K_{LL1}]\{T_{L}\left(t\right)\}-[K_{LP2}]\{Pv_{P}\left(t\right)\}-[K_{LL2}]\{Pv_{L}\left(t\right)\}-[K_{LP3}]\{P_{P}\left(t\right)\}-[K_{LL3}]\{P_{L}\left(t\right)\}+[C_{LL1}]\{∆Pv_{L}\}/∆t]$$

{*T_L_*}*^n^*^+1^ = {*T_L_*}*^n^* + {∆*T_L_* } (31)

(32) $$\{∆Pv_{L}\}[[C_{LL3}]+∆t\theta [K_{LL4}]]=∆t[-[K_{LP4}]\{Pv_{P}\left(t\right)\}-[K_{LL4}]\{Pv_{L}\left(t\right)\}-[K_{LP6}]\{T_{P}\left(t\right)\}-[K_{LL6}]\{T_{L}\left(t\right)\}-[K_{LP5}]\{P_{P}\left(t\right)\}-[K_{LL5}]\{P_{L}\left(t\right)\}]$$

{*Pv_L_*}*^n^*^+1^ = {*Pv_L_*}*^n^* + {∆*Pv_L_* } (33)

(34) $$\{∆P_{L}\}[[C_{LL4}]+∆t\theta [K_{LL5}]]=∆t[-[K_{LP5}]\{P_{P}\left(t\right)\}-[K_{LL5}]\{P_{L}\left(t\right)\}]$$

{*P_L_*}*^n^*^+1^ = {*P_L_*}*^n^* + {∆*P_L_* } (35)

**Figure 3:**
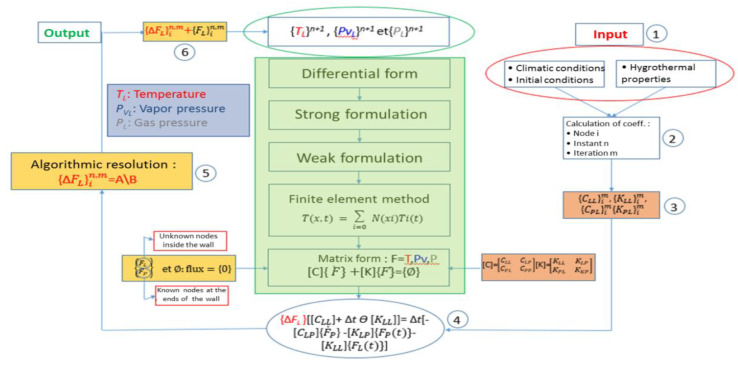
Algorithm for resolution of the transfer equations.

The algorithm in Figure 3 explains the overall resolution process.

### 3.3. Validation of the Chosen Heat, Air and Moisture (HAM) Model by the MATLAB Code

**Figure 4:**
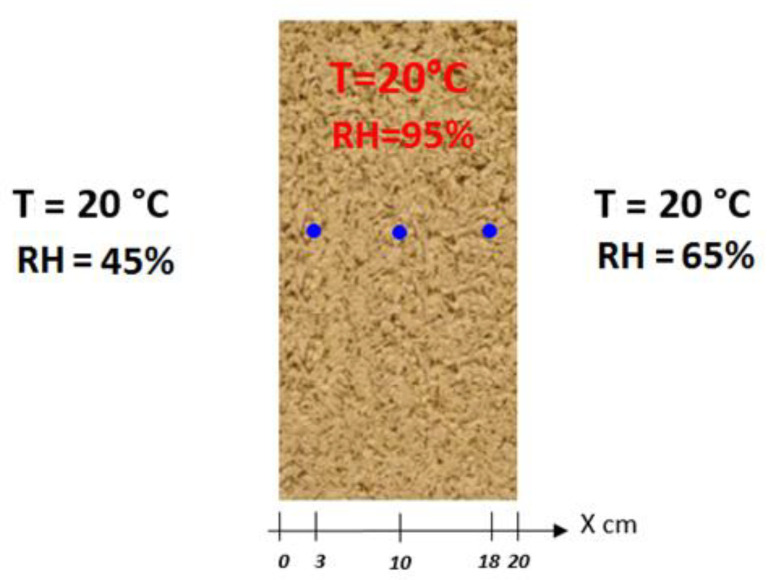
The international benchmark HAMSTAD WP2 study configuration [25].

In order to confidently use this model of prediction of hygrothermal transfers, it has been validated both analytically and experimentally. The benefit of this validation is to verify the accuracy of the numerical resolution offered by MATLAB. The results of the HAM model implemented in the MATLAB code are compared with the numerical results obtained by other HAM models, and with experimental results, based on an international benchmark HAMSTAD WP2 [25]. This test consists of determining the spatial distribution of the water content after 100, 300, and 1000 h. Initially, the 20 cm-thick wall is at equilibrium, with a relative humidity of 95% and a temperature of 20 °C. Maintaining the isothermal conditions, the wall is subjected to hydric stresses on both sides. The atmospheric relative humidity is reduced to 45% and the internal atmospheric relative humidity is set at 65%. Note that the vapor permeability, diffusion coefficient, and thermal conductivity are independent of the water content (Figure 4).

It is essential to validate the model for simpler cases by varying only one parameter (temperature or relative humidity) before performing experiments in cases with complex variation in relation to the boundary conditions. However, as heat and mass transfers are strongly coupled, we have performed our validation directly on a wall case where temperature and relative humidity vary simultaneously in order to get as close as possible to reality.

In [26], the hygrothermal behavior of a wall made of pre-cast HC blocks in a bi-climatic chamber was measured in different boundary conditions corresponding to realistic situations. The numerical model, once validated, was compared to a typical wall made of plain concrete, in the case of dynamic solicitation in temperature and vapor pressure on one side of the wall. The study highlighted the potential for the hemp-based wall to release moisture under an applied temperature difference, while water exchange with the ambient surroundings was prevented.

This validation confirms the robustness and accuracy of our one-dimensional HAM transfer simulation algorithm.

The properties of hemp concrete and the numerical parameters are presented in Table 1. The mesh size and the time step influence the accuracy of the numerical result. The smaller the time step, the more accurate the results. The adopted spatial mesh size and time step (∆t = 1 h and mesh size = 22 nodes) offer a stable solution. The input files and results for each exercise in the international HAMSTAD WP2 benchmark are available. Each file contains folders specifying the material properties and boundary conditions. In one case (exercise 2), the analytical solution is known and the parameters adopted in Table 1 are extracted from this test in order to validate the HAM model based on the MATLAB finite-element method.

**Figure 5:**
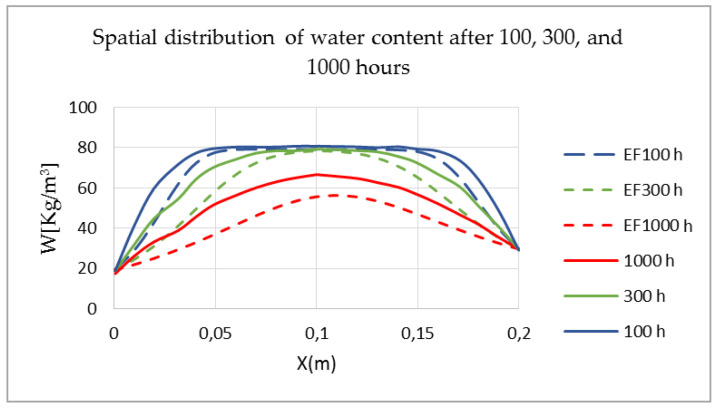
Spatial distribution of water content after 100, 300, and 1000 h [25].

The results of the chosen HAM model presented in Figure 5 by the dashed lines, at time 100 h and 300 h, exhibit satisfactory agreement with the numerical results of the international benchmark HAMSTAD WP2 [25] (shown by the solid line), except the difference at time 1000 h, which is due to an underestimation of the hydric storage capacity.

**Figure 6:**
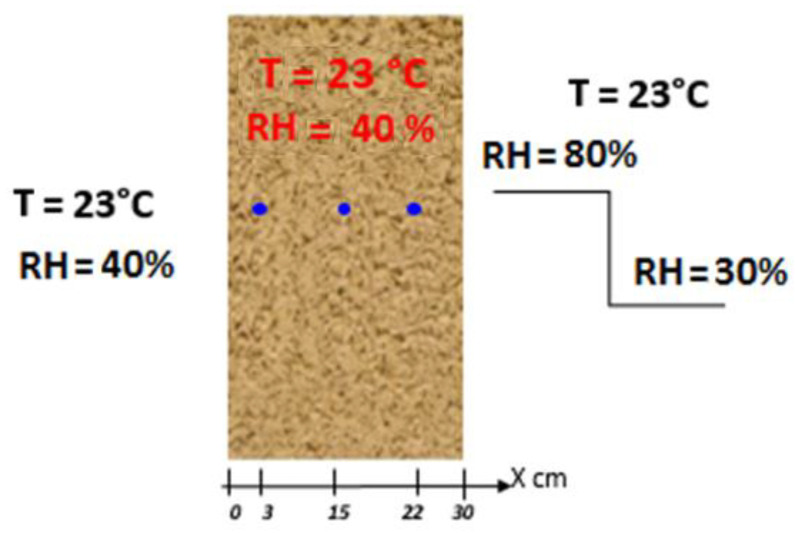
The study configuration proposed by [24].

For the experimental validation, we looked at the work of Oumeziane [24]. First, we present the study configuration in Figure 6—A 30 cm-thick hemp-concrete single-layer wall maintained initially at 23 °C and 40% RH, for 20 days. Therefore, the indoor conditions remain stable at 23 °C and 40% RH. The outdoor relative humidity set points are raised to 80%, but decrease sharply after 10 days to 30% RH. The temperature is always maintained at 23 °C. Note that the conditions throughout the campaign reflect recorded real-world atmospheric conditions, which oscillate significantly around the setpoint value. However, the numerical simulation conducted with the HAM model on MATLAB respects the exact set points shown in Figure 6.

By examining this humidity record, we are able to observe the behavior of hemp concrete subjected to solicitation in the adsorption and desorption phases.

**Figure 7:**
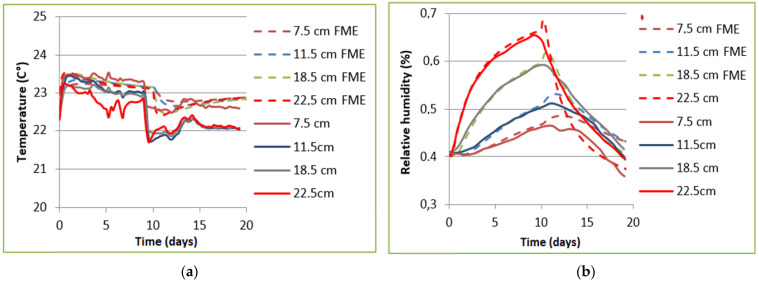
Comparison of temperature (T) (**a**) and relative humidity (RH) (**b**) of the numerical results obtained by the heat, air and moisture (HAM) model presented above and the experimental results [24].

Figure 7 presents the comparison of the numerical results obtained by the HAM model, computed by the finite element method on MATLAB, with the experimental results from the literature [24]. The numerical results show close agreement with the T and RH profiles obtained experimentally, during the adsorption phase. It seems, however, that the evolution of temperature and relative humidity during the desorption phase is less well reproduced than during the adsorption phase. Concerning the temperature distributions, a phase shift between numerical and experimental results is evident at all positions within the wall. This increase in temperature might be due to an underestimation of thermal dispersion with the outer atmosphere, leading to fluctuations in the evolution of RH within the wall due to the phase change effect. In the numerical approach, the Dirichlet boundary conditions are used. Hence, this approach does not take account of the surface exchanges or the quantities of heat and moisture actually entering and leaving the wall. Thus, it provides only a partial view of the hygrothermal behavior of the wall. This may also be due to an underestimation of the storage capacity in the desorption phase.

In conclusion, the HAM model described by the finite element method on MATLAB is able to predict the hygrothermal behavior of a hygroscopic wall subjected to stresses of T and RH on both sides.

## 4. Conclusions

Coupled HAM transfer through a porous building envelope has a major influence on energy consumption and indoor hygrothermal behavior. A literature review revealed divergences as to the numerical methods used to solve the classical hygrothermal transfer model. In addition, most programs do not offer full freedom of programming for model representation. In response to this situation, the current paper presented a phenomenological formulation for simulating HAM transfers through hemp concrete, considering temperature, water content, and total pressure gradient as driving potentials. The three non-linear coupled equations of the macroscopic model are based on the microscopic equations of mass and energy conservation. The discretized equations have been solved by the finite-element method using MATLAB. The numerical results show close agreement regarding the variation of the temperature and relative humidity profiles obtained experimentally, during the adsorption phase. It seems, however, that the evolution of temperature and relative humidity during the desorption phase is less well reproduced than that during the adsorption phase. This numerical simulation tool differs from others in that it offers a significant degree of programming freedom, in order to create a virtual representation of the model and achieve acceptable performance. The model exhibits good concordance with the one-dimensional numerical benchmark case No. 2 from the literature, and the experimental results obtained by Oumeziane [24]. This agreement demonstrates that the model implemented here is pertinent, and that the numerical resolution software (MATLAB) is efficient. The results show that the methodology adopted is robust and reliable to consider a rationalization of the model whose parameters are not ranked and not studied by their degree of influence.

One difficulty in the implementation of this transfer model lies in lack of knowledge of the intrinsic characteristics of the materials studied. In reality, these parameters are strongly interrelated but also variable. Thus, some parameters are often harder to measure analytically and experimentally. To address this problem, a sensitivity study must be undertaken to more precisely evaluate the influence of these parameters on the numerical response of a wall. Such a sensitivity analysis will highlight the parameters which have greatest influence on the hygrothermal transfers in building envelopes. From this point of view, to better understand the behavior of construction materials and improve the prediction of their hygrothermal performance, future work will develop a simplified numerical coupled HAM model based on a sensitivity analysis of certain parameters. The sensitivity analysis of the coupled HAM transfer model consists of changing one input parameter at a time. The output is compared to the reference case. The sensitivity analysis considers a distribution according to a probability density of the centered Gaussian law. On the other hand, this distribution is bounded and defined on a variation interval for each of the hygrothermal properties (input parameters). This approach is chosen because of the considerable disparity of the hygrothermal properties resulting from the morphological structure of hemp concrete. In this approach, the variation intervals of each input parameter are estimated on the basis of all the values found in the literature for bio-based materials. The model proposed in the present paper gives the freedom to vary these parameters with ease. It was also developed in such a way as to make the sensitivity analysis accessible and reliable.

## Figures and Tables

**Table 1:** Simulation input parameters for the N°2 HAMSTAD WP2 test [25].

Thickness	Mesh Size	∆*t*	Time	ρ	λ	*C_p_*	$K_{m}$	$C_{m}$
20 cm	22 nodes	1 h	1000 h	525 kg/m^3^	0.15 W/(m^2^K)	800 W/(m^2^K)	1.30 × 10^−11^ kg/(m.s.Pa)	4.13 × 10^−5^ kg/kg.Pa

## Data Availability

For the data supporting, please contact the corresponding author.
